# A stochastic Fubini theorem: BSDE method

**DOI:** 10.1186/s13660-017-1358-3

**Published:** 2017-04-14

**Authors:** Yanqing Wang

**Affiliations:** School of Mathematics and Statistics, Southwest University, Chongqing, 400715 China

**Keywords:** 60H05, 65C30, stochastic Fubini theorem, backward stochastic differential equation, random jumps

## Abstract

In this paper, we prove a stochastic Fubini theorem by solving a special backward stochastic differential equation (BSDE, for short) which is different from the existing techniques. As an application, we obtain the well-posedness of a class of BSDEs with the Itô integral in drift term under a subtle Lipschitz condition.

## Introduction and the main result

Given $T>0$, let $(\Omega,\mathcal{F},\mathcal{F}_{t},P;t\geq0)$ be a complete filtration space and $\mathbb{F}=\{\mathcal{F}_{t};{t \geq0}\}$ be a filtration satisfying the usual conditions which are generated by the following two mutually independent stochastic processes:

(i) a *d*-dimensional Brownian motion $\{B(t);t\geq0\}$;
(ii) a Poisson random measure *N* on $\mathbb{R}^{+}\times E$, where $E=\mathbb{R}^{l}-\{0\}$ with the Borel *σ*-field $\mathcal{B}(E)$. *λ* is the intensity (Lévy measure) of *N* with the property that
  $$\int_{E} \bigl(1\wedge \vert z\vert ^{2} \bigr) \lambda(dz)< \infty $$
  and *μ* is the compensator of *N* with $\mu(dt,dz)=dt\lambda(dz)$. Then $\{\widetilde{N}((0,t]\times A)=(N-\mu)((0,t]\times A), \mathcal{F}_{t};t\geq0\}$ is a compensated Poisson process which is a càdlàg martingale for all $A\in\mathcal{B}(E)$ satisfying $\lambda(A)<\infty$.

Fubini theorems giving conditions for change of the order of integration in multiple integrals are useful in all forms of calculus. The first such result of stochastic Fubini theorems perhaps belongs to Doob [1]. After that, there are two directions on this topic. One is to generalize the Itô integrals; the other is to study the theorem under the weaker integrability conditions (see [2–5] and the references therein). In those works, suppose that *M* is a stochastic process and $(X,\Sigma,\mu)$ is a *σ*-finite measure space, and $\phi: X\times[0,T]\times\Omega\rightarrow\mathbb{R}$ is a stochastic process satisfying certain measurability properties. Under some integrability conditions, the following stochastic Fubini theorem holds:

1.1 $$ \int_{X} \int_{0}^{t}\phi(x,s)\,dM(s)\,d\mu(x)= \int_{0}^{t} \int_{X} \phi(x,s)\,d\mu(x)\,dM(s), \quad t\in[0,T]. $$

The usual technique to prove (1.1) is the approximation method, i.e., firstly, (1.1) is proved for a simple process $\phi_{n}$ which is used to approximate *ϕ* in appropriate processes space, then it is proved by taking the appropriate limit. In this work, we treat a special case: $X=[0,T]$. In this case *ϕ* is only a process in $[0,T]$, and does the Fubini theorem hold? The key point is that the Lebesgue integral should be $\mathbb{F}$-adapted so that the Itô integral makes sense. In this paper, we want to prove this type of stochastic Fubini theorem by using the backward stochastic differential equation (BSDE, for short) method.

For simplicity, we consider only the case $d=l=1$ throughout this paper; the general cases can be treated by a similar method. For any $n\geq1$, denote by $\vert x\vert $ the Euclidean norm of $x\in\mathbb{R}^{n}$. Also, we define the following classes of processes which will be used in the sequel.

- For any $t\in[0,T]$, $L^{2}_{\mathcal{F}_{t}}(\Omega; \mathbb{R} ^{n})$ is the space of all $\mathcal{F}_{t}$-measurable and $\mathbb{R}^{n}$-valued random variables *ξ* satisfying $\vert \xi \vert ^{2} _{L^{2}_{\mathcal{F}_{t}}(\Omega; \mathbb{R}^{n})}=\mathbb{E}\vert \xi \vert ^{2}< \infty$.
- $L^{2}_{\mathbb{F}}(\Omega; D([0,T]; \mathbb{R}^{n}))$ is the space of all $\mathbb{F}$-adapted càdlàg stochastic processes $X(\cdot)$ satisfying $\vert X(\cdot)\vert ^{2}_{L^{2}_{\mathbb{F}}( \Omega; D([0,T]; \mathbb{R}^{n}))}=\mathbb{E} (\sup_{t\in [0,T]}\vert X(t)\vert ^{2} )<\infty$.
- $L^{2}_{\mathbb{F}}(\Omega; C([0,T]; \mathbb{R}^{n}))$ is the subspace of $L^{2}_{\mathbb{F}}(\Omega; D([0,T]; \mathbb{R}^{n}))$ whose element has continuous paths a.s.
- $L^{2}_{P,\mathbb{F}}(\Omega; L^{2}(0,T; \mathbb{R}^{n}))$ is the space of all $\mathbb{F}$-predictable and $\mathbb{R}^{n}$-valued stochastic processes $K(\cdot,\cdot)$ satisfying $\vert K(\cdot,\cdot)\vert ^{2} _{L^{2}_{P,\mathbb{F}}(\Omega; L^{2}(0,T; \mathbb{R}^{n}))}= \mathbb{E}\int_{0}^{T}\int_{E}\vert K(t,z)\vert ^{2}\lambda(dz)\,dt<\infty$.
- For general $p,q\geq1$, $L^{p}_{\mathbb{F}}(\Omega; L^{q}(0,T; \mathbb{R}^{n}))$ denotes the space of all $\mathbb{F}$-adapted processes $Y(\cdot)$ satisfying $\vert Y(\cdot)\vert ^{p}_{L^{p}_{ \mathbb{F}}(\Omega; L^{q}(0,T; \mathbb{R}^{n}))}=\mathbb{E} (\int _{0}^{T}\vert Y(t)\vert ^{q}\,dt)^{p/q}<\infty$.

The main theorem of this paper is stated as follows.

### Theorem 1.1

*For any* $Y(\cdot)\in L^{2}_{\mathbb{F}}(\Omega; L^{2}(0,T; \mathbb{R}^{n}))$, $K(\cdot,\cdot)\in L^{2}_{P,\mathbb{F}}(\Omega; L^{2}(0,T; \mathbb{R}^{n}))$ *and any* $g(\cdot),h(\cdot)\in L^{2}(0,T)$, *we have*

1.2 $$ \begin{aligned} & \int_{t}^{T}g(s) \int_{t}^{s}Y(u)\,dB(u)\,ds = \int_{t}^{T} \int_{s}^{T}g(u)\,du Y(s)\,dB(s), \\ & \int_{t}^{T}h(s) \int_{t}^{s} \int_{E}K(u,z)\widetilde{N}(du,dz)\,ds= \int_{t}^{T} \int_{E} \int_{s}^{T}h(u)\,duK(s,z)\widetilde{N}(ds,dz). \end{aligned} $$

As an application, under suitable conditions, we obtain the well-posedness of the following two BSDEs:

1.3 $$ \begin{aligned} y_{T}-y(t)= & \int_{t}^{T} \int_{t}^{s} f \bigl(u,Y(u) \bigr)\,dB(u)\,ds+ \int_{t}^{T}Y(s)\,dB(s),\quad \mbox{in } [0,T] \end{aligned} $$

and

1.4 $$ \begin{aligned} y_{T}-y(t)= & \int_{t}^{T} \int_{t}^{s} g \bigl(u,y(u),Y(u) \bigr)\,dB(u)\,ds+ \int _{t}^{T}Y(s)\,dB(s), \quad \mbox{in } [0,T]. \end{aligned} $$

The development of BSDEs theory has lasted for 40 years. Linear BSDEs were first introduced in 1973 by Bismut in [6] as the equations for the conjugate variable in the stochastic version of the Pontryagin maximum principle. Pardoux and Peng in [7] first studied the general nonlinear BSDEs of the following form in 1990:

1.5 $$ \begin{aligned} y_{T}-y(t)= \int_{t}^{T} l_{1} \bigl(s,y(s),Y(s) \bigr)\,ds+ \int_{t}^{T} l_{2} \bigl(s,y(s),Y(s) \bigr) \,dB(s), \quad \mbox{in } [0,T]. \end{aligned} $$

Since 1990, there has appeared a large number of works published related to the theory and applications for BSDEs (see [8–15] for examples).

It seems that (1.4) belongs to the following backward stochastic Volterra integral equation:

$$y(t)=k(t)- \int_{t}^{T}l \bigl(t,s,y(s),Y(t,s) \bigr)\,ds- \int_{t}^{T}Y(t,s)\,dB(s), \quad \mbox{in } [0,T], $$

which was studied in [15, 16]. Because of the special form, we can transform (1.3) and (1.4) into (1.5). Hence the second component *Y* of the solution just depends on one time variable.

The paper is organized as follows. In Section 2, we present some fundamental results, well-posedness of BSDEs and prove Theorem 1.1 by virtue of BSDEs. In Section 3, we apply Theorem 1.1 to solve BSDEs (3.1) and (3.2) and get the well-posedness under subtle Lipschitz conditions.

## Proof of the main result

The following BSDE with jump has been studied in some works, such as [11, 17]:

2.1 $$ \textstyle\begin{cases} dy(t)=f(t,y(t),Y(t),K(t,\cdot))\,dt+Y(t)\,dB(t)+\int_{E}K(t,z) \widetilde{N}(dt,dz),\quad \mbox{in } [0,T], \\ y(T)=y_{T}, \end{cases} $$

where *f* satisfies $f(\cdot,0,0,0)\in L^{2}_{\mathbb{F}}(\Omega; L ^{1}(0,T; \mathbb{R}^{n}))$, and

2.2 $$\begin{aligned}& \bigl\vert f(t,y,Y,K)-f(t, \widetilde{y}, \widetilde{Y},\widetilde{K}) \bigr\vert \\& \quad \leq L \bigl(\vert y- \widetilde{y}\vert +\vert Y-\widetilde{Y} \vert +\vert K-\widetilde{K} \vert _{L^{2}(E, \mathcal{B}(E),\lambda;\mathbb{R}^{n})} \bigr) \quad \mbox{a.e. } t\in[0,T], \mbox{a.s.}, \end{aligned}$$

for any $y, \widetilde{y}, Y, \widetilde{Y}\in\mathbb{R}^{n}$, $K, \widetilde{K}\in L^{2}(E,\mathcal{B}(E),\lambda;\mathbb{R}^{n})$.

The following lemma is about the well-posedness of (2.1). The proof can be found in [11, 17]. Hence, it is omitted. For simplicity, we denote $\mathcal{H}=L^{2}_{\mathbb{F}}(\Omega; D([0,T]; \mathbb{R}^{n}))\times L^{2}_{\mathbb{F}}(\Omega; L^{2}(0,T; \mathbb{R}^{n}))\times L^{2}_{P,\mathbb{F}}(\Omega; L^{2}(0,T; \mathbb{R}^{n}))$ with the canonical norm.

### Lemma 2.1

*For any* $y_{T}\in L^{2}_{\mathcal{F}_{T}}(\Omega;\mathbb{R}^{n})$, *equation* (2.1) *admits a unique adapted solution* $(y(\cdot),Y(\cdot),K(\cdot,\cdot))\in\mathcal{H}$. *Furthermore*, *there is a constant* $C>0$, *depending only on* *L* *and* *T*, *such that*

2.3 $$\begin{aligned}& \bigl\vert \bigl(y(\cdot),Y(\cdot),K(\cdot,\cdot) \bigr) \bigr\vert _{\mathcal{H}} \leq C \bigl( \bigl\vert f(\cdot,0,0,0) \bigr\vert _{L^{2}_{\mathbb{F}}(\Omega;L^{1}(0,T; \mathbb{R}^{n}))}+\vert y_{T}\vert _{L^{2}_{\mathcal{F}_{T}}(\Omega; \mathbb{R}^{n})} \bigr). \end{aligned}$$

In order to prove Theorem 1.1, we first consider the following BSDE in $[0,T]$:

2.4 $$\begin{aligned} y_{T}-y(t) = & \int_{t}^{T} \biggl(g(s) \int_{t}^{s} Y(u)\,dB(u)+h(s) \int _{t}^{s} \int_{E}K(u,z)\widetilde{N}(du,dz) \biggr)\,ds \\ &{}+ \int_{t}^{T}Y(s)\,dB(s)+ \int_{t}^{T} \int_{E}K(s,z)\widetilde{N}(ds,dz). \end{aligned}$$

$g(\cdot)$ and $h(\cdot)\in L^{1}(0,T)$ such that for any $t\in[0,T]$,

2.5 $$ \int_{t}^{T}g(s)\,ds+1>\delta,\qquad \int_{t}^{T}h(s)\,ds+1>\delta, $$

where *δ* is a positive constant.

The well-posedness of (2.4) is presented in the following theorem.

### Theorem 2.2

*Under assumption* (2.5), *for any* $y_{T}\in L^{2} _{\mathcal{F}_{T}}(\Omega;\mathbb{R}^{n})$, *equation* (2.4) *admits a unique adapted solution* $(y(\cdot),Y(\cdot),K(\cdot,\cdot))\in\mathcal{H}$ *such that*

2.6 $$ \bigl\vert \bigl(y(\cdot),Y(\cdot),K(\cdot,\cdot) \bigr)\bigr\vert _{\mathcal{H}} \leq C\vert y _{T}\vert _{L^{2}_{\mathcal{F}_{T}}(\Omega;\mathbb{R}^{n})}, $$

*where* *C* *is a constant depending on* *δ* *and* *T*.

### Proof

We divide the proof into two steps.

**Step 1.** By Lemma 2.1, we know that the following BSDE

2.7 $$\begin{aligned}& y_{T}-y(t) \\& \quad = \int_{t}^{T} \biggl( \int_{s}^{T}g(u)\,du+1 \biggr)Y(s)\,dB(s) \\& \qquad {}+ \int_{t}^{T} \int_{E} \biggl( \int_{s}^{T}h(u)\,du+1 \biggr)K(s,z) \widetilde{N}(ds,dz) \end{aligned}$$

admits a unique solution $(y(\cdot),Y(\cdot),K(\cdot,\cdot))$ satisfying

2.8 $$ \biggl\vert \biggl(y(\cdot), \biggl( \int_{\cdot}^{T}g(u)\,du+1 \biggr)Y(\cdot), \biggl( \int_{\cdot}^{T}h(u)\,du+1 \biggr)K(\cdot,\cdot) \biggr) \biggr\vert _{ \mathcal{H}} \leq C\vert y_{T}\vert _{L^{2}_{\mathcal{F}_{T}}(\Omega; \mathbb{R}^{n})}, $$

where *C* depends only on *T*. Hence, by virtue of (2.5), it follows that

2.9 $$\begin{aligned}& \begin{aligned} & \bigl\vert Y(\cdot) \bigr\vert \leq1/\delta \biggl\vert \biggl( \int_{\cdot}^{T}g(u)\,du+1 \biggr)Y(\cdot) \biggr\vert , \\ & \bigl\vert K(\cdot,\cdot) \bigr\vert \leq1/\delta \biggl\vert \biggl( \int_{\cdot}^{T}h(u)\,du+1 \biggr)K(\cdot,\cdot) \biggr\vert . \end{aligned} \end{aligned}$$

Then, by (2.8) and (2.9), we deduce that

2.10 $$ \bigl\vert \bigl(y(\cdot),Y( \cdot),K(\cdot,\cdot) \bigr) \bigr\vert _{\mathcal{H}} \leq C\vert y_{T}\vert _{L^{2}_{\mathcal{F}_{T}}(\Omega;\mathbb{R}^{n})}, $$

where *C* depends on *δ* and *T*.

**Step 2.** By (2.7), we can easily obtain

2.11 $$\begin{aligned} y(t)-y(0) =& \int_{0}^{t} \biggl( \int_{s}^{T}g(u)\,du+1 \biggr)Y(s)\,dB(s) \\ &{}+ \int _{0}^{t} \int_{E} \biggl( \int_{s}^{T}h(u)\,du+1 \biggr)K(s,z) \widetilde{N}(ds,dz). \end{aligned}$$

Applying Itô’s formula to $(\int_{t}^{T}g(s)\,ds+1)\int _{0}^{t}Y(u)\,dB(u)$, one can get

2.12 $$\begin{aligned}& \int_{0}^{t} \biggl( \int_{s}^{T}g(u)\,du+1 \biggr)Y(s)\,dB(s) \\& \quad = \biggl( \int _{t}^{T}g(s)\,ds+1 \biggr) \int_{0}^{t}Y(u)\,dB(u)+ \int_{0}^{t}g(s) \int_{0} ^{s}Y(u)\,dB(u)\,ds. \end{aligned}$$

Similarly, one has

2.13 $$\begin{aligned}& \int_{0}^{t} \int_{E} \biggl( \int_{s}^{T}h(u)\,du+1 \biggr)K(s,z) \widetilde{N}(ds,dz) \\& \quad = \biggl( \int_{t}^{T}h(s)\,ds+1 \biggr) \int_{0}^{t} \int_{E}K(u,z)\widetilde{N}(du,dz) \\& \qquad {}+ \int_{0}^{t}h(s) \int_{0}^{s} \int _{E}K(u,z)\widetilde{N}(du,dz)\,ds. \end{aligned}$$

Substituting (2.12) and (2.13) into (2.7) yields

2.14 $$\begin{aligned}& y(t)-y(0) \\& \quad = \int_{0}^{t}g(s) \int_{0}^{s}Y(u)\,dB(u)\,ds+ \int_{0}^{t}h(s) \int_{0}^{s} \int_{E}K(u,z)\widetilde{N}(du,dz)\,ds \\& \qquad {}+ \biggl( \int_{t} ^{T}g(s)\,ds+1 \biggr) \int_{0}^{t}Y(u)\,dB(u)+ \biggl( \int_{t}^{T}h(s)\,ds+1 \biggr) \int_{0}^{t} \int_{E}K(u,z)\widetilde{N}(du,dz) \\& \quad = \int_{0}^{t}g(s) \int_{0}^{s}Y(u)\,dB(u)\,ds+ \int_{0}^{t}h(s) \int_{0}^{s} \int_{E}K(u,z) \widetilde{N}(du,dz)\,ds \\& \qquad {}+ \int_{t}^{T}g(s)\,ds \int_{0}^{t}Y(u)\,dB(u)+ \int_{t}^{T}h(s)\,ds \int_{0}^{t} \int_{E}K(u,z)\widetilde{N}(du,dz) \\& \qquad {}+ \int_{0}^{t}Y(s)\,dB(s)+ \int_{0}^{t} \int_{E}K(s,z)\widetilde{N}(ds,dz) \\& \quad = \biggl( \int_{0}^{t}g(s) \int_{0}^{s}+ \int_{t}^{T}g(s) \int_{0}^{t}+ \int_{t}^{T}g(s) \int_{t}^{s}- \int_{t}^{T}g(s) \int_{t}^{s} \biggr)Y(u)\,dB(u)\,ds \\& \qquad {}+ \biggl( \int_{0}^{t}h(s) \int_{0}^{s}+ \int_{t}^{T}h(s) \int_{0}^{t}+ \int_{t}^{T}h(s) \int_{t}^{s}- \int_{t}^{T}h(s) \int_{t}^{s} \biggr) \int _{E}K(u,z)\widetilde{N}(du,dz)\,ds \\& \qquad {}+ \int_{0}^{t}Y(s)\,dB(s)+ \int_{0} ^{t} \int_{E}K(s,z)\widetilde{N}(ds,dz) \\& \quad = \biggl( \int_{0}^{T}g(s) \int _{0}^{s}- \int_{t}^{T}g(s) \int_{t}^{s} \biggr) Y(u)\,dB(u)\,ds \\& \qquad {}+ \biggl( \int _{0}^{T}h(s) \int_{0}^{s}- \int_{t}^{T}h(s) \int_{t}^{s} \biggr) \int_{E}K(u,z) \widetilde{N}(du,dz)\,ds \\& \qquad {}+ \int_{0}^{t}Y(s)\,dB(s)+ \int_{0}^{t} \int_{E}K(s,z) \widetilde{N}(ds,dz). \end{aligned}$$

Taking $t=T$ in (2.14), one has

2.15 $$\begin{aligned} y_{T}-y(0) = & \int_{0}^{T}g(s) \int_{0}^{s} Y(u)\,dB(u)\,ds+ \int_{0}^{T}h(s) \int_{0}^{s} \int_{E}K(u,z)\widetilde{N}(du,dz)\,ds \\ &{}+ \int_{0}^{T}Y(s)\,dB(s)+ \int_{0}^{T} \int_{E}K(s,z)\widetilde{N}(ds,dz). \end{aligned}$$

Subtracting (2.14) from (2.15), we just obtain (2.4). Hence $(y(\cdot),Y(\cdot),K( \cdot,\cdot))$ is a unique solution to (2.4). The desired estimate (2.6) follows from (2.10). That completes the proof. □

As a corollary, we give the proof of the stochastic Fubini theorem stated in Theorem 1.1.

### Proof of Theorem 1.1

Set

2.16 $$\begin{aligned} \xi = & \int_{0}^{T} \biggl(g^{+}(s) \int_{0}^{s} Y(u)\,dB(u)+h^{+}(s) \int _{0}^{s} \int_{E}K(u,z)\widetilde{N}(du,dz) \biggr)\,ds \\ &{}+ \int_{0}^{T}Y(s)\,dB(s)+ \int_{0}^{T} \int_{E}K(s,z)\widetilde{N}(ds,dz), \end{aligned}$$

where $g^{+}(\cdot)$, $h^{+}(\cdot)$ are the positive parts of $g(\cdot)$, $h(\cdot)$, respectively. By Hölder’s inequality and the Itô isometry, we have

2.17 $$\begin{aligned}& \vert \xi \vert ^{2}_{L^{2}_{\mathcal{F}_{T}}(\Omega;\mathbb{R}^{n})} \\ & \quad \leq 4 \biggl(T\mathbb{E} \int_{0}^{T} \bigl(g^{+}(s) \bigr)^{2} \biggl( \int _{0}^{s}Y(u)\,dB(u) \biggr)^{2}\,ds \\ & \qquad {}+T \mathbb{E} \int_{0}^{T} \bigl(h^{+}(s) \bigr)^{2} \biggl( \int_{0}^{s} \int_{E}K(u,z)\widetilde{N}(ds,dz) \biggr)^{2}\,ds \\ & \qquad {}+\vert Y\vert ^{2}_{L^{2}_{\mathbb{F}}(\Omega; L^{2}(0,T; \mathbb{R}^{n}))}+\vert K\vert ^{2} _{L^{2}_{P,\mathbb{F}}(\Omega; L^{2}(0,T; \mathbb{R}^{n}))} \biggr) \\ & \quad \leq4 \bigl(T \bigl\vert g^{+} \bigr\vert ^{2}_{L^{2}(0,T)}+1 \bigr)\vert Y\vert ^{2}_{L^{2}_{ \mathbb{F}}(\Omega; L^{2}(0,T; \mathbb{R}^{n}))}+4 \bigl(T \bigl\vert h^{+} \bigr\vert ^{2} _{L^{2}(0,T)}+1 \bigr)\vert K \vert ^{2}_{L^{2}_{P,\mathbb{F}}(\Omega; L^{2}(0,T; \mathbb{R}^{n}))} \\ & \quad < \infty. \end{aligned}$$

By the proof of Theorem 2.2, it is easy to get that

2.18 $$\begin{aligned} y(T)-y(t) = & \int_{t}^{T} \biggl(g^{+}(s) \int_{t}^{s} Y(u)\,dB(u)+h^{+}(s) \int_{t}^{s} \int_{E}K(u,z)\widetilde{N}(du,dz) \biggr)\,ds \\ &{}+ \int_{t} ^{T}Y(s)\,dB(s)+ \int_{t}^{T} \int_{E}K(s,z)\widetilde{N}(ds,dz), \quad \mbox{in } [0,T], \end{aligned}$$

with terminal condition $y(T)=\xi$ admits a unique solution $(y(\cdot),Y(\cdot),K(\cdot,\cdot))$. And (2.18) is equivalent to

2.19 $$\begin{aligned} y(T)-y(t) =& \int_{t}^{T} \biggl( \int_{s}^{T}g^{+}(u)\,du+1 \biggr)Y(s) \,dB(s) \\ &{}+ \int_{t}^{T} \int_{E} \biggl( \int_{s}^{T}h^{+}(u)\,du+1 \biggr)K(s,z) \widetilde{N}(ds,dz). \end{aligned}$$

Combining (2.18) and (2.19), we can get

2.20 $$\begin{aligned}& \begin{aligned} & \int_{t}^{T}g^{+}(s) \int_{t}^{s}Y(u)\,dB(u)\,ds= \int_{t}^{T} \int_{s} ^{T}g^{+}(u)\,du Y(s)\,dB(s), \\ & \int_{t}^{T}h^{+}(s) \int_{t}^{s} \int_{E}K(u,z) \widetilde{N}(du,dz)\,ds= \int_{t}^{T} \int_{E} \int_{s}^{T}h^{+}(u)\,duK(s,z) \widetilde{N}(ds,dz). \end{aligned} \end{aligned}$$

Similarly, we have

2.21 $$\begin{aligned}& \begin{aligned} & \int_{t}^{T}g^{-}(s) \int_{t}^{s}Y(u)\,dB(u)\,ds = \int_{t}^{T} \int_{s}^{T}g ^{-}(u)\,du Y(s)\,dB(s), \\ & \int_{t}^{T}h^{-}(s) \int_{t}^{s} \int_{E}K(u,z) \widetilde{N}(du,dz)\,ds= \int_{t}^{T} \int_{E} \int_{s}^{T}h^{-}(u)\,duK(s,z) \widetilde{N}(ds,dz). \end{aligned} \end{aligned}$$

Addition of (2.20) and (2.21) gives (1.2), which completes the proof. □

## An application: well-posedness of two BSDEs

In this section, we consider only the BSDEs driven by one-dimensional Brownian motions. The other cases such as BSDEs driven by high dimension Brownian motions and BSDEs with jumps can also be treated in a similar procedure. Let $(\Omega,\mathcal{F},\mathcal{F}_{t},P;t\geq0)$ be a complete filtration space and $B(\cdot)$ be a one-dimensional standard Brownian motion whose natural filtration is given by $\mathbb{F}=\{ \mathcal{F}_{t}\}_{t\geq0}$. As an application of Theorem 1.1, we prove the well-posedness of the following two BSDEs:

3.1 $$ \begin{aligned} y_{T}-y(t)= & \int_{t}^{T} \int_{t}^{s} f \bigl(u,Y(u) \bigr)\,dB(u)\,ds+ \int_{t}^{T}Y(s)\,dB(s), \quad \mbox{in } [0,T] \end{aligned} $$

and

3.2 $$ \begin{aligned} y_{T}-y(t)= & \int_{t}^{T} \int_{t}^{s} g \bigl(u,y(u),Y(u) \bigr)\,dB(u)\,ds+ \int _{t}^{T}Y(s)\,dB(s),\quad \mbox{in } [0,T]. \end{aligned} $$

Here, generators $f(\cdot,\cdot)$ and $g(\cdot,\cdot,\cdot)$ satisfy the following assumptions:

1. $f(\cdot,0)\in L^{2}_{\mathbb{F}}(\Omega; L^{2}(0,T; \mathbb{R}^{n}))$,
  $$\bigl\vert f(t,Y)-f(t,\widetilde{Y}) \bigr\vert \leq\frac{1}{T+\theta} \vert Y-\widetilde{Y}\vert \quad \mbox{a.e. } t\in[0,T], \mbox{a.s.}, $$
  and
2. $g(\cdot,0,0)\in L^{2}_{\mathbb{F}}(\Omega; L^{2}(0,T; \mathbb{R}^{n}))$,
  $$\bigl\vert g(t,y,Y)-g(t,\widetilde{y},\widetilde{Y}) \bigr\vert \leq L \vert y-\widetilde{y}\vert +\frac{1}{2(T+ \theta)}\vert Y-\widetilde{Y}\vert \quad \mbox{a.e. } t \in[0,T], \mbox{a.s.}, $$

respectively, where $y, \widetilde{y}, Y, \widetilde{Y}\in \mathbb{R}^{n}$, and *L* and *θ* are positive constants.

### Theorem 3.1

*Under assumption* (H1), *for any* $y_{T}\in L^{2}_{\mathcal{F} _{T}}(\Omega;\mathbb{R}^{n})$, *equation* (3.1) *admits a unique adapted solution* $(y(\cdot),Y(\cdot))\in L^{2}_{\mathbb{F}}(\Omega; C([0,T]; \mathbb{R}^{n}))\times L^{2}_{\mathbb{F}}(\Omega; L^{2}(0,T; \mathbb{R}^{n}))$ *such that*

3.3 $$\begin{aligned}& \bigl\vert \bigl(y(\cdot),Y(\cdot) \bigr) \bigr\vert _{L^{2}_{\mathbb{F}}(\Omega; C([0,T]; \mathbb{R}^{n}))\times L^{2}_{\mathbb{F}}(\Omega; L^{2}(0,T; \mathbb{R}^{n}))} \\& \quad \leq C \bigl(\vert y_{T}\vert _{L^{2}_{\mathcal{F}_{T}}( \Omega;\mathbb{R}^{n})}+ \bigl\vert f( \cdot,0) \bigr\vert _{L^{2}_{\mathbb{F}}(\Omega; L ^{2}(0,T; \mathbb{R}^{n}))} \bigr), \end{aligned}$$

*where* *C* *is a constant depending on* *θ* *and* *T*.

### Proof

We divide the proof into three steps.

**Step 1.** Suppose that $f(\cdot,Y(\cdot))\in L^{2}_{ \mathbb{F}}(\Omega; L^{2}(0,T; \mathbb{R}^{n}))$. Then by Theorem 1.1 we rewrite equation (3.1) as follows:

3.4 $$ y_{T}-y(t)= \int_{t}^{T} \bigl((T-s)f \bigl(s,Y(s) \bigr)+Y(s) \bigr)\,dB(s),\quad \mbox{in } [0,T]. $$

We know that the following BSDE admits a unique adapted solution $(y(\cdot), Z(\cdot))$

3.5 $$ \textstyle\begin{cases} dy(t)=Z(t)\,dB(t),\quad \mbox{in } [0,T], \\ y(T)=y_{T}. \end{cases} $$

We define an operator $S: L^{2}_{\mathbb{F}}(\Omega; L^{2}(0,T; \mathbb{R}^{n}))\rightarrow L^{2}_{\mathbb{F}}(\Omega; L^{2}(0,T; \mathbb{R}^{n}))$ by $S(X(t))=Z(t)- (T-t)f(t,X(t))$ for a.e. *t*. For any $X_{1}, X_{2}\in L^{2}_{\mathbb{F}}(\Omega; L^{2}(0,T; \mathbb{R} ^{n}))$ and a.e. *t*,

$$\begin{aligned} \bigl\vert S \bigl(X_{1}(t) \bigr)-S \bigl(X_{2}(t) \bigr) \bigr\vert =&(T-t) \bigl\vert f \bigl(t,X_{1}(t) \bigr)-f \bigl(t,X_{1}(t) \bigr) \bigr\vert \\ \leq&(T-t)/(T+\theta) \bigl\vert X_{1}(t)-X_{2}(t) \bigr\vert \\ \leq& T/(T+\theta) \bigl\vert X_{1}(t)-X_{2}(t) \bigr\vert . \end{aligned}$$

We see that *S* is contractive. Hence, by the Banach fixed point theorem, *S* admits a unique fixed point $Y(\cdot)\in L^{2}_{ \mathbb{F}}(\Omega; L^{2}(0,T; \mathbb{R}^{n}))$ such that $S(Y(\cdot))=Y(\cdot)$, i.e.,

3.6 $$ (T-t)f \bigl(Y(t) \bigr)+Y(t)=Z(t)\quad \mbox{a.e. } t \in[0,T]. $$

By (3.5) and (3.6), we conclude that $(y(\cdot), Y(\cdot))$ is the unique solution to (3.1).

**Step 2.** In this step, we check that for this $Y(\cdot)$, $f(\cdot,Y(\cdot))\in L^{2}_{\mathbb{F}}(\Omega; L^{2}(0,T; \mathbb{R}^{n}))$

$$\begin{aligned}& \bigl\vert f \bigl(\cdot,Y(\cdot) \bigr) \bigr\vert ^{2}_{L^{2}_{\mathbb{F}}(\Omega; L^{2}(0,T; \mathbb{R}^{n}))} \\& \quad \leq 2 \bigl\vert f \bigl(\cdot,Y(\cdot) \bigr)-f(\cdot,0) \bigr\vert ^{2}_{L ^{2}_{\mathbb{F}}(\Omega; L^{2}(0,T; \mathbb{R}^{n}))}+2T \bigl\vert f(\cdot,0) \bigr\vert ^{2} _{L^{2}_{\mathbb{F}}(\Omega; L^{2}(0,T; \mathbb{R}^{n}))} \\& \quad \leq 2/(T+ \theta)\vert Y\vert ^{2}_{L^{2}_{\mathbb{F}}(\Omega; L^{2}(0,T; \mathbb{R} ^{n}))}+2T \bigl\vert f(\cdot,0) \bigr\vert ^{2}_{L^{2}_{\mathbb{F}}(\Omega; L^{2}(0,T; \mathbb{R}^{n}))} \\& \quad < \infty. \end{aligned}$$

**Step 3.** In this step, we show that (3.3) holds.

By Jensen’s inequality, Doob’s maximal inequality and equation (3.5), we see that

3.7 $$ \mathbb{E} \Bigl\vert \sup_{t\in[0,T]}y(t) \Bigr\vert ^{2}=\mathbb{E} \Bigl\vert \sup_{t\in[0,T]} \mathbb{E}(y_{T}\vert \mathcal{F}_{t}) \Bigr\vert ^{2}\leq4 \mathbb{E}\vert y_{T}\vert ^{2}. $$

Applying Itô’s formula to $\vert y(\cdot)\vert ^{2}$, we get

3.8 $$ \mathbb{E} \int_{0}^{T} \bigl\vert (T-t)f \bigl(t,Y(t) \bigr)+Y(t) \bigr\vert ^{2}\,dt\leq\mathbb{E}\vert y_{T} \vert ^{2}. $$

On the other hand, one has

3.9 $$\begin{aligned}& \mathbb{E} \int_{0}^{T} \bigl\vert (T-t)f \bigl(t,Y(t) \bigr)+Y(t) \bigr\vert ^{2}\,dt \\& \quad \geq\mathbb{E} \int_{0}^{T} \bigl( \bigl\vert (T-t) \bigl(f \bigl(t,Y(t) \bigr)-f(t,0) \bigr)+Y(t) \bigr\vert - \bigl\vert (T-t)f(t,0) \bigr\vert \bigr)^{2}\,dt \\& \quad \geq\frac{1}{2}\mathbb{E} \int_{0}^{T} \bigl\vert (T-t) \bigl(f \bigl(t,Y(t) \bigr)-f(t,0) \bigr)+Y(t) \bigr\vert ^{2}\,dt -3\mathbb{E} \int_{0}^{T} \bigl\vert (T-t)f(t,0) \bigr\vert ^{2}\,dt. \end{aligned}$$

By $\vert a+b\vert ^{2}\geq(1-\varepsilon)\vert a\vert ^{2}+1-1/\varepsilon \vert b\vert ^{2}$, where $\varepsilon>1$, and assumption (H1), we can obtain

3.10 $$\begin{aligned}& \mathbb{E} \int_{0}^{T} \bigl\vert (T-t) \bigl(f \bigl(t,Y(t) \bigr)-f(t,0) \bigr)+Y(t) \bigr\vert ^{2}\,dt \\& \quad \geq(1-\varepsilon)\mathbb{E} \int _{0}^{T} \bigl\vert (T-t) \bigl(f \bigl(t,Y(t) \bigr)-f(t,0) \bigr) \bigr\vert ^{2}\,dt+(1-1/ \varepsilon)\mathbb{E} \int_{0}^{T} \bigl\vert Y(t) \bigr\vert ^{2}\,dt \\& \quad \geq(1-\varepsilon)\mathbb{E} \int_{0}^{T} \biggl(\frac{T-t}{T+\theta} \biggr)^{2} \bigl\vert Y(t) \bigr\vert ^{2}\,dt+ \biggl(1- \frac{1}{\varepsilon} \biggr)\mathbb{E} \int_{0}^{T} \bigl\vert Y(t) \bigr\vert ^{2}\,dt \\& \quad \geq \biggl[(1-\varepsilon) \biggl(\frac{T}{T+\theta} \biggr)^{2}+ \biggl(1-\frac{1}{\varepsilon} \biggr) \biggr]\mathbb{E} \int _{0}^{T} \bigl\vert Y(t) \bigr\vert ^{2}\,dt. \end{aligned}$$

Taking $\varepsilon=\frac{T+\theta}{T}$, by (3.10), we have

3.11 $$ \mathbb{E} \int_{0}^{T} \bigl\vert (T-t) \bigl(f \bigl(t,Y(t) \bigr)-f(t,0) \bigr)+Y(t) \bigr\vert ^{2}\,dt \geq \biggl( \frac{\theta}{T+\theta} \biggr)^{2}\mathbb{E} \int_{0}^{T} \bigl\vert Y(t) \bigr\vert ^{2}\,dt. $$

(3.11), together with (3.9), yields

3.12 $$\begin{aligned}& \mathbb{E} \int_{0}^{T} \bigl\vert (T-t)f \bigl(t,Y(t) \bigr)+Y(t) \bigr\vert ^{2}\,dt \\& \quad \geq\frac{1}{2} \biggl( \frac{\theta}{T+\theta} \biggr)^{2} \mathbb{E} \int_{0}^{T} \bigl\vert Y(t) \bigr\vert ^{2}\,dt -3T^{2}\mathbb{E} \int_{0}^{T} \bigl\vert f(t,0) \bigr\vert ^{2}\,dt. \end{aligned}$$

Combining (3.8) and (3.12), one has

3.13 $$ \bigl\vert Y(\cdot) \bigr\vert ^{2}_{L^{2}_{\mathbb{F}}(\Omega; L^{2}(0,T; \mathbb{R} ^{n}))}\leq C \bigl(\mathbb{E}\vert y_{T} \vert ^{2}+ \bigl\vert f(\cdot,0) \bigr\vert ^{2}_{L^{2}_{ \mathbb{F}}(\Omega; L^{2}(0,T; \mathbb{R}^{n}))} \bigr), $$

where *C* is a constant depending on *θ* and *T*. That completes the proof. □

We list two examples from which we can see that the Lipschitz constant $1/(T+\theta)$ cannot be improved.

### Example 3.2

*For any* $\theta>0$, *suppose that* $y_{T}=\int_{\theta}^{T}(s- \theta)/(T-\theta)\,dB(s)$ *and* $f(s,x)=-\chi_{[0,\theta]}(s)x/(T-s)- \chi_{(\theta,T]}(s)x/(T-\theta)$ *with Lipschitz constant* $1/(T-\theta)$. *Then equation* (3.1) *can be written as*

$$y_{T}-y(t)= \int_{t}^{T}(s-\theta)/(T-\theta) \chi_{(\theta,T]}(s)Y(s)\,dB(s), \quad \textit{in } [0,T]. $$

*For any* $\eta\in L^{2}_{\mathbb{F}}((0,\theta)\times\Omega; \mathbb{R})$, *let*

$$\begin{aligned}& y(t) =\chi_{(\theta,T]}(t) \int_{\theta}^{t}(s-\theta)/(T-\theta)\,dB(s), \\& Y_{\eta}(t)=\chi_{[0,\theta]}(t)\eta(t)+\chi_{(\theta,T]}(t),\quad \forall t\in[0,T]. \end{aligned}$$

*It is easy to check that* $(y(\cdot),Y_{\eta}(\cdot))\in L^{2}_{ \mathbb{F}}(\Omega;C([0,T];\mathbb{R}))\times L^{2}_{\mathbb{F}}( \Omega;L^{2}(0,T;\mathbb{R}))$ *is an adapted solution of* (3.1). *Since* *η* *is arbitrary*, *the solution of* (3.1) *is not unique*.

### Example 3.3

*Set* $y_{T}=B(T)$ *and* $f(t,x)=-x/T$ *whose Lipschitz constant is* $1/T$. *If equation* (3.1) *has an adapted solution* $(y,Y)\in L^{2}_{\mathbb{F}}(\Omega;C([0,T];\mathbb{R})) \times L^{2}_{\mathbb{F}}(\Omega;L^{2}(0,T;\mathbb{R}))$, *then by Theorem* 1.1, (3.1) *becomes*

3.14 $$ y_{T}-y(t)= \int_{t}^{T}\frac{s}{T}Y(s)\,dB(s). $$

*Since* $(B(\cdot),1)$ *is the unique adapted solution of* $y_{T}-y(t)= \int_{t}^{T}Z(t)\,dB(t)$, *we get that*

$$Y(t)=T/tZ(t)\chi_{\{t\neq0\}}(t)=T/t\chi_{\{t\neq0\}}(t). $$

*It is clear that* $\vert Y(\cdot)\vert _{ L^{2}_{\mathbb{F}}(\Omega;L^{2}(0,T; \mathbb{R}))}=+\infty$. *Hence* (3.1) *with* $f(t,x)=-x/T, y_{T}=B(T)$ *does not have a square integrable adapted solution*.

From the previous two examples, we know that in equation (3.1), Lipschitz constant $1/(T+\theta)$ cannot be improved. But in the one-dimensional case, we can obtain a better result.

### Theorem 3.4

*Suppose that in equation* (3.1), $y_{T}\in L^{2}_{\mathcal{F}_{T}}(\Omega;\mathbb{R})$, $f(\cdot,0) \in L^{2}_{\mathbb{F}}(\Omega; L^{2}(0,T; \mathbb{R}))$, *and*

$$1/(T+\theta) (x_{1}-x_{2})\leq f(\cdot, x_{1})-f(\cdot, x_{2}) \leq L(x_{1}-x_{2}), \quad \forall x_{1}\geq x_{2}, $$

*where* *θ* *and* *L* *are positive constants and* $L\geq1/(T+\theta)$. *Then BSDE* (3.1) *admits a unique solution*.

### Proof

Set $h(t,x)=(T-t)f(t,x)+x$. It is easy to see that

$$\theta/(T+\theta) (x_{1}-x_{2})\leq h(t,x_{1})-h(t,x_{2}) \leq(TL+1) (x _{1}-x_{2}), \quad \forall x_{1} \geq x_{2}. $$

By Theorem 4.1 in [7], we conclude that (3.1) admits a unique solution. □

For $\beta>0$ and $X\in L^{2}_{\mathbb{F}}(\Omega; L^{2}(0,T; \mathbb{R}^{n}))$, $\Vert X\Vert _{\beta}^{2}$ denotes $\mathbb{E}\int _{0}^{T}e^{\beta t}\vert X(t)\vert ^{2}\,dt$. $H_{\beta}$ denotes the space $L^{2}_{\mathbb{F}}(\Omega; L^{2}(0,T; \mathbb{R}^{n}))$ endowed with the norm $\Vert \cdot \Vert _{\beta}$.

### Theorem 3.5

*Under assumption* (H2), *for any* $y_{T}\in L^{2}_{\mathcal{F} _{T}}(\Omega;\mathbb{R}^{n})$, *equation* (3.2) *admits a unique adapted solution* $(y(\cdot),Y(\cdot))$ *such that*

3.15 $$\begin{aligned}& \bigl\vert \bigl(y(\cdot),Y(\cdot) \bigr) \bigr\vert _{L^{2}_{\mathbb{F}}(\Omega; C([0,T]; \mathbb{R}^{n}))\times L^{2}_{\mathbb{F}}(\Omega; L^{2}(0,T; \mathbb{R}^{n}))} \\& \quad \leq C \bigl(\vert y_{T}\vert _{L^{2}_{\mathcal{F}_{T}}( \Omega;\mathbb{R}^{n})}+ \bigl\vert g( \cdot,0,0) \bigr\vert _{L^{2}_{\mathbb{F}}(\Omega; L^{2}(0,T; \mathbb{R}^{n}))} \bigr), \end{aligned}$$

*where* *C* *is a constant depending on* *L* *and* *T*.

### Proof

We divide the proof into two steps.

**Step 1.** For any $\bar{y}(\cdot),\bar{Y}(\cdot)\in L^{2} _{\mathbb{F}}(\Omega; L^{2}(0,T; \mathbb{R}^{n}))$, by virtue of the martingale representation theorem, the following BSDE admits a unique solution $(y(\cdot), Y(\cdot))$

3.16 $$ y_{T}-y(t)= \int_{t}^{T} \int_{t}^{s} g \bigl(u,\bar{y}(u),\bar{Y}(u) \bigr) \,dB(u)\,ds+ \int_{t}^{T}Y(s)\,dB(s),\quad \mbox{in } [0,T]. $$

**Step 2.** We use the Banach fixed point theorem for the mapping Φ from $H_{\beta}\times H_{\beta}$ to itself, which maps $(\bar{y}(\cdot),\bar{Y}(\cdot))$ onto $(y(\cdot),Y(\cdot))$, where *β* is determined later.

For any $(\bar{y}_{i}(\cdot),\bar{Y}_{i}(\cdot))$, suppose that the corresponding solution to (3.16) is $(y_{i}(\cdot),Y_{i}(\cdot))$, $i=1,2$.

In order to use Theorem 1.1, we first check that $g(\cdot,\bar{y_{i}}(\cdot),\bar{Y_{i}}(\cdot))\in L^{2}_{ \mathbb{F}}(\Omega; L^{2}(0,T; \mathbb{R}^{n}))$. Indeed, applying the same method as that in Step 2 of Theorem 3.1, one has

$$\begin{aligned}& \bigl\vert g \bigl(t,\bar{y_{i}}(t), \bar{Y_{i}}(t) \bigr) \bigr\vert ^{2}_{L^{2}_{\mathbb{F}}(\Omega; L^{2}(0,T; \mathbb{R}^{n}))} \\& \quad \leq 2 \bigl\vert g \bigl(t,\bar{y_{i}}(t), \bar{Y_{i}}(t) \bigr)-g(t,0,0) \bigr\vert ^{2}_{L^{2}_{\mathbb{F}}(\Omega; L^{2}(0,T; \mathbb{R}^{n}))}+2 \bigl\vert g( \cdot,0,0) \bigr\vert _{L^{2}_{\mathbb{F}}(\Omega; L^{2}(0,T; \mathbb{R}^{n}))} \\& \quad \leq 4L^{2} \bigl\vert \bar{y_{i}}(\cdot) \bigr\vert ^{2} _{L^{2}_{\mathbb{F}}(\Omega; L^{2}(0,T; \mathbb{R}^{n}))}+1/(T+ \theta)^{2} \bigl\vert \bar{Y_{i}}(\cdot) \bigr\vert ^{2}_{L^{2}_{\mathbb{F}}(\Omega; L ^{2}(0,T; \mathbb{R}^{n}))} \\& \qquad {}+2 \bigl\vert g(\cdot,0,0) \bigr\vert _{L^{2}_{\mathbb{F}}( \Omega; L^{2}(0,T; \mathbb{R}^{n}))} \\& \quad < \infty. \end{aligned}$$

Hence, by Theorem 1.1, for $i=1, 2$, (3.16) turns to

3.17 $$ y_{i}(T)-y_{i}(t)= \int_{t}^{T} \bigl((T-s)g \bigl(s, \bar{y_{i}}(s),\bar{Y _{i}}(s) \bigr)+Y_{i}(s) \bigr)\,dB(s),\quad \mbox{in } [0,T]. $$

Set $\Delta y=y_{1}-y_{2}$, $\Delta Y=Y_{1}-Y_{2}$, $\Delta\bar{y}= \bar{y}_{1}-\bar{y}_{2}$, $\Delta\bar{Y}=\bar{Y}_{1}-\bar{Y}_{2}$ and $\Delta g(\cdot)=g(\cdot,\bar{y}_{1}(\cdot),\bar{Y}_{1}(\cdot))-g( \cdot,\bar{y}_{2}(\cdot),\bar{Y}_{2}(\cdot))$. Applying Itô’s formula to $e^{\beta\cdot}(y_{1}(\cdot)-y_{2}(\cdot))^{2}$, we have

$$\begin{aligned}& e^{\beta T} \bigl(\Delta y(T) \bigr)^{2}-e^{\beta t} \bigl( \Delta y(t) \bigr)^{2} \\& \quad =\beta \int_{t}^{T}e^{\beta s} \bigl(\Delta y(s) \bigr)^{2}\,ds+2 \int_{t}^{T}e^{\beta s} \bigl( \Delta y(s) \bigr) \bigl[(T-s) \bigl(\Delta g(s) \bigr)+ \bigl(\Delta Y(s) \bigr) \bigr] \,dB(s) \\& \qquad {}+ \int_{t}^{T} e ^{\beta s} \bigl[(T-s) \bigl( \Delta g(s) \bigr)+ \bigl(\Delta Y(s) \bigr) \bigr]^{2}\,ds. \end{aligned}$$

Setting $y_{1}(T)=y_{2}(T)$ and taking expectation, we get

$$\begin{aligned}& \mathbb{E}e^{\beta t} \bigl(\Delta y(t) \bigr)^{2}+\beta \mathbb{E} \int_{t}^{T}e ^{\beta s} \bigl(\Delta y(s) \bigr)^{2}\,ds+\mathbb{E} \int_{t}^{T}e^{\beta s} \bigl( \Delta Y(s) \bigr)^{2}\,ds \\& \quad =-\mathbb{E} \int_{t}^{T}e^{\beta s}(T-s)^{2} \bigl( \Delta g(s) \bigr)^{2}\,ds-2\mathbb{E} \int_{t}^{T} e^{\beta t}(T-s)\Delta g(s) \Delta Y(s)\,ds, \quad \forall t\in[0,T]. \end{aligned}$$

Let $t=0$,

$$\begin{aligned}& \beta \Vert \Delta y\Vert ^{2}_{\beta}+ \Vert \Delta Y \Vert ^{2}_{\beta} \\& \quad \leq 2 \mathbb{E} \int_{0}^{T} e^{\beta s}(T-s) \biggl(L\Delta \bar {y}(s)+\frac{1}{2(T+ \theta)}\Delta\bar{Y}(s) \biggr)\Delta Y(s)\,ds \\& \quad < \varepsilon_{1}L ^{2}\mathbb{E} \int_{0}^{T} e^{\beta s}(T-s)^{2} \bigl(\Delta\bar {y}(s) \bigr)^{2}\,ds+\frac{1}{ \varepsilon_{1}}\Vert \Delta Y \Vert _{\beta}^{2} \\& \qquad {}+\frac{\varepsilon_{2}}{4(T+ \theta)^{2}}\mathbb{E} \int_{0}^{T} e^{\beta s}(T-s)^{2} \bigl(\Delta \bar{Y}(s) \bigr)^{2}\,ds+\frac{1}{\varepsilon_{2}}\Vert \Delta Y \Vert _{\beta}^{2} \\& \quad \leq\varepsilon_{1}L^{2}T^{2}\Vert \Delta \bar{y}\Vert _{\beta}^{2}+\frac{ \varepsilon_{2}T^{2}}{4(T+\theta)^{2}} \Vert \Delta \bar{Y}\Vert _{\beta} ^{2}+ \biggl(\frac{1}{\varepsilon_{1}}+ \frac{1}{\varepsilon_{1}} \biggr)\Vert \Delta Y\Vert _{\beta}^{2}, \end{aligned}$$

which is equivalent to

$$\Vert \Delta y\Vert ^{2}_{\beta}+ \frac{ (1-\frac{1}{\varepsilon _{1}}-\frac{1}{ \varepsilon_{1}} )}{\beta} \Vert \Delta Y\Vert ^{2}_{\beta} < \frac{ \varepsilon_{1}L^{2}T^{2}}{\beta} \biggl(\Vert \Delta\bar{y}\Vert _{\beta} ^{2}+\frac{\varepsilon_{2}}{4\varepsilon_{1}L^{2}(T+\theta)^{2}} \Vert \Delta\bar{Y}\Vert _{\beta}^{2} \biggr). $$

Take $\varepsilon_{1}$, $\varepsilon_{2}$ and *β* satisfying

3.18 $$ \textstyle\begin{cases} \varepsilon_{1}>1,\qquad \varepsilon_{2}>1,\qquad \frac{1}{\varepsilon_{1}}+\frac{1}{\varepsilon_{1}}< 1, \\ \frac{ (1-\frac{1}{\varepsilon_{1}}-\frac{1}{\varepsilon_{1}} )}{\beta}> \frac{\varepsilon_{2}}{4\varepsilon _{1}L^{2}(T+\theta)^{2}}, \\ \frac{\varepsilon_{1}L^{2}T^{2}}{\beta}< 1. \end{cases} $$

We know these $\varepsilon_{1}$, $\varepsilon_{2}$ and *β* exist. For example, one can take $\varepsilon_{1}=\frac{2(1+\alpha)}{ \alpha}$, $\varepsilon_{2}=2$, and $\beta=\frac{2\varepsilon_{1}L ^{2}T^{2}(T+\theta)^{2}}{(T+\theta)^{2}+T^{2}}$, where $\alpha =\frac{2T\theta+\theta^{2}}{4T^{2}}$. For this *β*, we see that Φ is a contraction and that there exists a fixed point, which is the unique solution of BSDE (3.2).

By the same method used in Step 3 of Theorem 3.1, we can show that (3.15) holds. That completes the proof. □

## Conclusions

In this paper, we consider a stochastic Fubini theorem with two time variables. In that Fubini theorem, one time variable is related to the Lebesgue integral, and the other is related to the Itô integral, in which the adapted property is crucial. We prove the result by solving a BSDE which is different from the existing method. As an application, we apply the theorem to study the well-posedness of two special BSDEs under subtle Lipschitz conditions. Besides this, we provide two examples to state that aforementioned Lipschitz conditions cannot be improved.

## References

[1] Doob JL (1953). Stochastic Processes.

[2] Bichteler K, Lin SJ (1995). On the stochastic Fubini theorem. Stoch. Stoch. Rep..

[3] Kailath T, Segall A, Zakai M (1978). Fubini-type theorems for stochastic integrals. Sankhya, Ser. A.

[4] León JA (1990). Stochastic Fubini theorem for semimartingales in Hilbert space. Can. J. Math..

[5] van Neerven J, Veraar MC (2006). On the stochastic Fubini theorem in infinite dimensions. Stochastic Partial Differential Equations and Applications - VII.

[6] Bismut JM (1973). Conjugate convex functions in optimal stochastic control. J. Math. Anal. Appl..

[7] Pardoux É, Peng S (1990). Adapted solution of a backward stochastic differential equation. Syst. Control Lett..

[8] El Karoui N, Huang SJ (1997). A general result of existence and uniqueness of backward stochastic differential equations. Backward Stochastic Differential Equations (Paris, 1995–1996).

[9] El Karoui N, Peng S, Quenez MC (1997). Backward stochastic differential equations in finance. Math. Finance.

[10] Li J (2006). Fully coupled forward-backward stochastic differential equations with general martingale. Acta Math. Sci. Ser. B Engl. Ed..

[11] Tang SJ, Li XJ (1994). Necessary conditions for optimal control of stochastic systems with random jumps. SIAM J. Control Optim..

[12] Wang Y (2013). BSDEs with general filtration driven by Lévy processes, and an application in stochastic controllability. Syst. Control Lett..

[13] Wang Y, Zhang C (2015). The norm optimal control problem for stochastic linear control systems. ESAIM Control Optim. Calc. Var..

[14] Wu Z (1999). Forward-backward stochastic differential equations with Brownian motion and Poisson process. Acta Math. Appl. Sinica (Engl. Ser.).

[15] Yong J (2008). Well-posedness and regularity of backward stochastic Volterra integral equations. Probab. Theory Relat. Fields.

[16] Lin J (2002). Adapted solution of a backward stochastic nonlinear Volterra integral equation. Stoch. Anal. Appl..

[17] Barles G, Buckdahn R, Pardoux E (1997). Backward stochastic differential equations and integral-partial differential equations. Stoch. Stoch. Rep..

